# Resilience of Healthcare Systems in the face of COVID-19: an experience report

**DOI:** 10.1590/1980-220X-REEUSP-2021-0210en

**Published:** 2022-05-27

**Authors:** Ariane Ranzani Rigotti, Cristina Mara Zamarioli, Patrícia Rezende do Prado, Flávia Helena Pereira, Fernanda Raphael Escobar Gimenes

**Affiliations:** 1 Universidade de São Paulo, Escola de Enfermagem de Ribeirão Preto, Ribeirão Preto, SP, Brazil.; 2 Universidade Federal do Acre, Rio Branco, AC, Brazil.; 3 Instituto Federal de Educação Ciências e Tecnologia do Sul de Minas Gerais, Passos, MG, Brazil.

**Keywords:** Coronavirus Infections, Resilience, Psychological, Patient Safety, Health Systems, Health Personnel, Infecciones por Coronavirus, Resiliencia Psicológica, Seguridad del Paciente, Sistemas de Salud, Personal de Salud, Infecções por Coronavírus, Resiliência Psicológica, Segurança do Paciente, Sistemas de Saúde, Pessoal de Saúde

## Abstract

**Objective:**

to report the professional experience of a nurse manager facing the challenges of restructuring a hospital service in the face of the COVID-19 pandemic.

**Method:**

this is an experience report, based on the perspective of system resilience in a public hospital.

**Results:**

the challenges faced were: internal service flow reorganization to assist suspected cases of COVID-19; institution of structural changes and adaptations, from entry into the emergency room to the wards and intensive care unit; equipment and supply acquisition for patient care with a focus on their quality and functionality; staff training, with the restructuring of work processes; staff sizing, considering the time of exposure to the virus; staff’s professional qualification, absenteeism, stress, physical and psychological illness, with a view to safe and quality care; nursing staff leadership to deal with conflicts generated by professionals’ stress and illness.

**Conclusion:**

healthcare service resilience is critical for hospital restructuring in the COVID-19 pandemic; however, patient care and healthcare professionals’ physical and mental health must be considered.

## RESUMO

**Objetivo:**

relatar a experiência profissional de um enfermeiro gestor frente aos desafios da reestruturação de um serviço hospitalar diante da pandemia da COVID-19.

**Método:**

relato de experiência, baseado na perspectiva da resiliência do sistema em um hospital público.

**Resultados:**

os desafios enfrentados foram: reorganização do fluxo de serviço interno para atender os casos suspeitos de COVID-19; instituição de mudanças e adaptações estruturais, desde a entrada na urgência e emergência, até nas enfermarias e unidade de terapia intensiva; aquisição de equipamentos e insumos para o atendimento dos pacientes com foco na qualidade e funcionalidade destes; treinamento das equipes, com a reestruturação dos processos de trabalho; dimensionamento de pessoal, considerando o tempo de exposição ao vírus; qualificação profissional, absenteísmo, estresse, adoecimento físico e psicológico da equipe, com vistas à uma assistência segura e de qualidade; liderança da equipe de Enfermagem para lidar com os conflitos gerados pelo estresse e adoecimento dos profissionais.

**Conclusão:**

a resiliência do serviço de saúde é fundamental para a reestruturação hospitalar na pandemia da COVID-19, no entanto, devem-se considerar o cuidado dos pacientes e a saúde física e mental dos profissionais de saúde.

## RESUMEN

**Objetivo:**

relatar la experiencia profesional de una enfermera gestora frente a los desafíos de la reestructuración de un servicio hospitalario frente a la pandemia de la COVID-19.

**Método:**

relato de experiencia, basado en la perspectiva de resiliencia del sistema en un hospital público.

**Resultados:**

los desafíos enfrentados fueron: reorganización del flujo interno de atención para la atención de casos sospechosos de COVID-19; institución de cambios y adaptaciones estructurales, desde el ingreso al departamento de urgencias y emergencias hasta las salas y unidad de cuidados intensivos; adquisición de equipos e insumos para la atención de pacientes con enfoque en su calidad y funcionalidad; formación de equipos con la reestructuración de los procesos de trabajo; dimensionamiento del personal, considerando el tiempo de exposición al virus, cualificación profesional, ausentismo, estrés, enfermedad física y psíquica del equipo, con miras a un cuidado seguro y de calidad y liderazgo del equipo de enfermería para el enfrentamiento de los conflictos generados por el estrés y la enfermedad de los profesionales.

**Conclusión:**

la resiliencia del servicio de salud es fundamental para la reestructuración hospitalaria en la pandemia del COVID-19, sin embargo, se debe considerar la atención al paciente y la salud física y mental de los profesionales de la salud.

## INTRODUCTION

On March 11, 2020, the World Health Organization (WHO) declared COVID-19 a pandemic due to the increase in the number of cases outside China and the number of countries affected by the new coronavirus^(1)^. On February 3, 2020, there was no record of confirmed disease cases and deaths in Brazil, however on March 30, 2020, the WHO recorded an increase of 5,639 confirmed cases of the disease and 267 deaths in Brazil^(2)^.

System resilience refers to the ability to absorb disturbances, respond to them, and recover when exposed to external threats. Resilient systems develop the ability to adapt and positively transform their structures and means of operation to provide the required service^(3)^.

Managing a pandemic requires a robust hospital structure that demonstrates fast and assertive decision-making to control the spread of the virus^(4)^. From this perspective, the COVID-19 pandemic has challenged Healthcare Systems (HCS) on a global scale and, in Brazil, this scenario required the Unified Health System (SUS – *Sistema Único de Saúde*) to also adapt to face the crisis^(5)^. However, the weaknesses presented by SUS, aggravated by the political and economic crisis and the conduct of the federal government, were evident^(6)^.

Furthermore, the COVID-19 pandemic has also demonstrated that HCS are essential both in stabilizing the right to health and in maintaining social and economic services. Therefore, countries that were successful in facing the pandemic will be more prepared for the return of social and economic activities, even if these are regularized after the population is vaccinated^(6)^.

Equipping HCS with administrative, staffing and structural reserve capacity in pandemic situations will require creative approaches, including the provision of an “army of healthcare providers” that can be quickly mobilized, the ability to reserve supplies such as Personal Protective Equipment (PPE) and the maintenance of hospital beds, which can be quickly transformed into intensive care beds^(7)^.

Given the scenario, this study aimed to report the professional experience of a nurse manager facing the challenges of restructuring a hospital service in the face of the COVID-19 pandemic.

## METHOD

### Design of Study

This experience report is based on the perspective of system resilience^(3)^.

### Local

The study was carried out in a Brazilian high school university hospital that offers medical-surgical care to adult patients from 19 municipalities located in the countryside of the state of São Paulo.

Before the COVID-19 pandemic, the hospital had ten intensive care beds for adults and 156 inpatient beds, which were intended for SUS and supplementary care. Each year, the hospital treated around 6,000 patients, with approximately 450,000 outpatient visits and an average occupancy rate of 76%. In the operating room, on average, 152 small and medium-sized surgeries were performed per month. The medical-surgical care wards, which later began to provide assistance to patients with acute respiratory distress syndrome (ARDS), had 45 beds.

The institution was prepared to care for up to 12 critical patients, as it had 18 mechanical ventilators, excluding those intended for surgery, 30 multi-parameter monitors and 15 cardioverters, the latter being allocated according to Brazilian legal aspects^(8)^. The hospital had 228 nursing professionals. Of these, only 24 nursing technicians and five nurses were qualified to work in caring for critically ill patients, whose assistance refers to immediate clinical or surgical care with intensive support of equipment and supplies.

Training for the correct PPE use and hand hygiene practices followed an annual schedule that was planned by the Permanent Education Center (PEC) and the Hospital Infection Control Commission (HICC). Generally, these trainings were carried out once a semester and/or according to the institution’s employees’ specific needs, inpatient units and other sectors.

In February 2020, the planning of actions to face the spread of COVID-19 began after the Ministry of Health declared a public health emergency of national concern^(9)^. From then on, transformations in the structure and processes were initiated. Weekly meetings with the institution’s management staff were necessary. A crisis committee was created whose objectives were to align decisions on the health staff needed to care for patients with COVID-19 and plan the budget for acquiring the new equipment and supplies necessary to face the pandemic.

During the COVID-19 pandemic, the institution became a reference hospital and health staff had to prepare themselves to carry out the diagnosis and treatment of patients with COVID-19, according to the protocols proposed by the WHO and the Ministry of Health.

The nurse manager, for playing the role of leadership and being responsible for most of the health staff, which is necessary for caring for suspected and confirmed cases of COVID-19, can actively participate in decisions with the institution’s Management Committee. Therefore, the nursing manager faced several challenges related to the nursing staff, which will be discussed in the results.

### Ethical Aspects

As this is an experience report, the study did not require approval from the Research Ethics Committee. However, authorization was requested for disclosure of data and images of the service to the institution’s administration.

## RESULTS

Through the experience report, the main challenges faced by the nurse manager were described, established in five categories: *Institution’s internal service flow reorganization*; *Equipment and supply acquisition for care*; *Staff training*; *Staff sizing*; and *Challenges in the nursing staff leadership process*.

### Institution’s Internal Service Flow Reorganization

**Figure 1:**
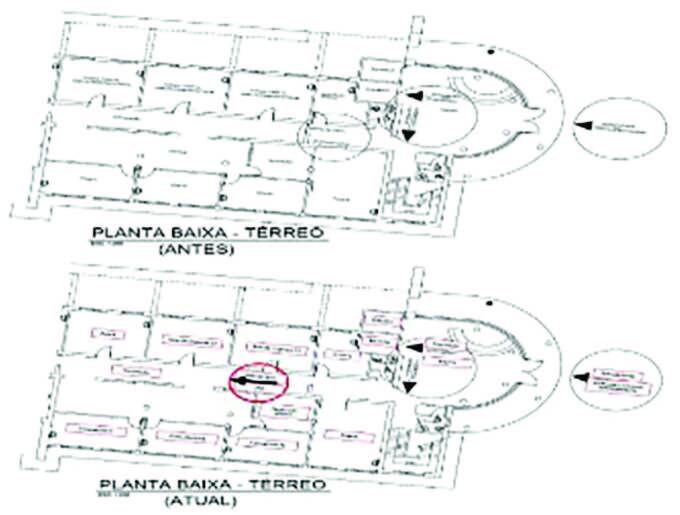
New flowchart of care for patients suspected or with COVID-19 on the ground floor. TRANSLATION: waiting room; office 01/02/03; room 01/02/03/04; closed door; open door; medical prescription; entrance; exit; X-ray; archives; emergency room 01/02; elevator; inpatient unit; ARU; care; flow; private area; health insurance area; SUS; isolation; lower floor plan – ground (before); lower floor plan – ground (current); reception; gynecology/obstetrics office 01/02/03; first floor; doctor’s room.

Source: Catanduva, SP, Brazil, 2020.

The first change took place from February to March 2020 and aimed to reorganize the care flow for patients with suspected COVID-19 infection. The objective of this movement was to guarantee a quality and safe service to all those involved. Figure 1 shows the internal and external flowchart of inpatients, before and after reorganization.

Aspects related to safety of health staff during the provision of care to suspected patients and those with COVID-19 were considered, inpatient and outpatient safety, safety of family members and maintenance of care provided to non-COVID patients were considered. Then, the transfer of patients hospitalized in beds intended exclusively for caring for suspected or confirmed cases of COVID-19 infection to other wards was planned.

**Figure 2.**
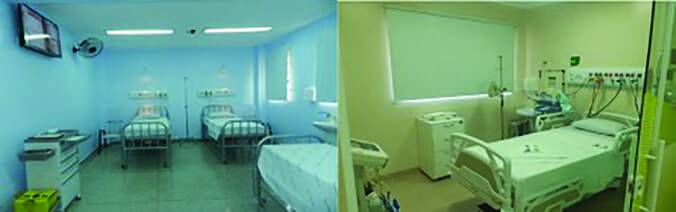
Units intended for caring for non-critical patients (ward on the left) and critical patients (ICU on the right) suspected and/or with COVID-19.

Note: the strategy aimed at the proximity between the ward and ICU beds.

Source: Catanduva, SP, Brazil, 2020.

These changes required structural and process adaptations, including increasing the number of ICU beds in the wards. Thus, 20 more beds were made available for caring for critically ill patients and another 22 beds for caring for patients classified as non-critical, totaling 208 beds in the hospital, as shown in Figure 2.

Outpatient care, at this stage, was restricted to high-risk pregnant women, orthopedics and emergency consultations previously scheduled by the municipality. The gynecology and obstetrics outpatient clinic, due to its link with the sectors of care for patients with ARDS, was restructured and transformed into an emergency room, which today is called Acute Respiratory Unit (ARU), whose objective was to manage primary care and clinical triage of patients with symptoms of respiratory distress. Soon, the ARU began to function as a “gateway” to the service for suspected patients with COVID-19. Therefore, emergency beds, medical offices and satellite pharmacy were included in this new unit.

**Figure 3.**
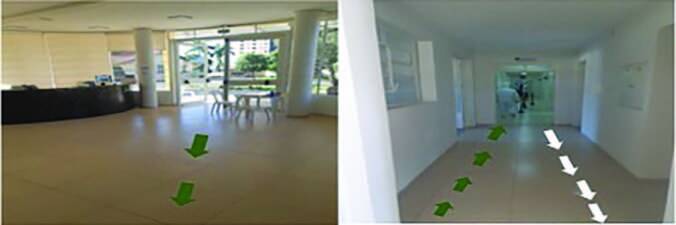
Internal care flow reorganization for patients suspected or with COVID-19. Note: the green arrows indicate the entry of patients through the ARU for medical care and the white arrows, the exit after medical discharge.

Source: Catanduva, SP, Brazil, 2020.

The care unit for patients with suspected or confirmed COVID-19 was isolated from the other hospital units and sectors by means of physical barriers, drywall-type plaster walls, as it is a quick-installation and low-cost material. This change was essential to make the new flow clear and safe for all stakeholders, indicating the inflow and outflow of patients by means of arrows (Figure 3).

Due to the adjustments made, there was a 70% reduction in surgical procedures, from 152 elective surgeries to 42 monthly surgeries in this period. Consequently, there was also a 19% reduction in the hospital occupancy rate, with a total of 62% of beds occupied, compared to the months prior to April 2020.

### Equipment and Supply Acquisition for Care

Another challenge faced was the need to structure ARDS patient care sectors with equipment and supplies; however, there were not many options available on the market. However, the management staff focused on each item’s quality and functionality and not on the cost, as the focus was on providing quality and safety assistance for all those involved. For equipment and supply acquisition, the institution’s own resources in the amount of US$ 378,947.37 were invested. The new mechanical ventilators were quickly acquired by the hospital’s administrative management and, therefore, there were no difficulties in acquiring 14 pieces of equipment.

Regarding the other equipment, the institution provided two cardioverters, two defibrillators and 15 multiparameter monitors. However, until June 10, 2020, there was no delivery forecast, with the need to relocate monitors from other units, as well as from another hospital, to assist the first cases of patients with COVID-19.

PPE unavailability in the national and international market contributed to the deficit in the institution. However, after training with the staff on rational use, based on WHO and Ministry of Health guidelines, consumption was reduced and this challenge was overcome.

### Staff Training

Faced with the changes that were necessary in the structure, material and equipment acquisition, work processes also needed to be reorganized, and there is a need for specific training of health staff for safe and quality care for patients and health professionals. The trainings followed an intense schedule and were directed to the practice of hand hygiene with soap and water and preparations with 70% alcohol; safety in putting on and taking off aprons; the correct PPE use at each stage of care; proper disposal of PPE; ARDS patient care based on the best available evidence; notification of suspected and confirmed cases and information management. As new equipment arrived at the institution, training was carried out with all health professionals for the safe and rational use.

All frontline healthcare providers were trained, considering the need for future health staff relocation to care for patients with confirmed or suspected COVID-19 in one of the peak phases of the disease in the country and, especially, in the region. HICC, PEC and the nursing coordination also carried out training with all professionals in the hospital administration sector and support services, such as nutrition and dietetics, laboratory, pharmacy, image, hygiene and cleaning.

The training lasted one week and occurred daily, with an average duration of 40 minutes each. The training was carried out during the working period, did not generate overtime and there was compliance by healthcare providers. Interest and curiosity, as it is a new and still unknown disease, contributed to 100% of professionals being trained without difficulties.

### Staff Sizing

One of the greatest challenges, in the process of restructuring the institution into a reference hospital in the region for caring for patients with COVID-19, was the nursing staff sizing. Because it is a disease still little known, with controversial clinical management protocols, nursing coordinators experienced feelings of anguish, concern and fear, with the staff tending to develop Burnout Syndrome. During this period, there was an increase in the number of medical certificates and absences from work that impacted nursing professionals’ daily and monthly schedule. In addition to this, the need to develop new staff schedules and dimensions on a daily basis was already perceptible. Thus, caution was necessary to select the nursing staff that would provide care to patients.

There was concern about the criteria that would be adopted in this selection in order to promote patient and worker safety. One of the challenges for managing this process was the lack of qualified personnel to provide emergency care to patients and emotionally prepared to face the crisis. Previous experience in caring for critically ill patients with ARDS was a criterion established in the selection process, in addition to complying with COFEN Resolution, referring to the minimum number of nursing professionals, for assistance within 24 hours, considering the degree of dependence of patients^(10)^.

Healthcare providers’ double working hours were also considered. Therefore, those who worked in care units for COVID-19 patients at other health institutions were not selected, aiming to reduce the risk of developing Burnout Syndrome, which could impact quality of care and professional and patient safety.

These criteria were enough to make the nursing staff sizing even more challenging, since the institution already had high turnover rates of nursing professionals before the pandemic.

There was a need to reallocate healthcare providers from another hospital to compose the staff, considering emergency and critical care. The difficulty in operationalizing staff sizing, according to the criteria defined for the pandemic period, was noticed during the development of the first schedule, and it was necessary to include healthcare providers that did not fit the intended profile. Furthermore, nursing assistants were also included in the monthly staff sizing, in order to guarantee the minimum health staff essential for caring for patients with suspected COVID-19. In order to prepare them to face the pandemic, administrative shifts were created in the area of COVID-19. A nursing managerial supervisor was available 24 hours a day to monitor the care flow and assistance provided to patients, as well as to support decision-making.

### Challenges in the Nursing Staff Leadership Process

During the period of physical and organizational restructuring, the health service was challenged to reorganize itself to deal with the conflicts generated by the entire nursing staff’s stress and illness. The institution was concerned with adopting specific assistance strategies aimed at assisting employees who started flu-like symptoms. The protocol, at first, consisted of a flowchart for outpatient care with due scheduling for medical assessment and rapid testing to confirm the disease. If so, the service offered medical follow-up until the nursing professional was able to return to their activities in the work environment 15 days after the onset of the first symptoms.

In addition to physical illness, some nursing professionals showed emotional fragility in the face of the stress experienced by the emergence of a new virus at a time of ignorance of the means of disease transmission and treatment. Moreover, those who already had mental illnesses had these exacerbated during this period.

On the other hand, given this scenario, the institution had not developed, within the contingency plan, a service aimed at the nursing staff’s mental illness. Nursing management, during this period, did not receive support from administrative management in order to deal with the conflict generated by the psychological illness of professionals. Even with the management being directed to be concerned with staff sizing, avoiding work overload and exposure to the unknown pathogen, there was no training and administrative support to devise strategies on how to deal with professionals’ psychological illness and presenteeism, with the purpose of maintaining a safe service and environment for patients and the nursing staff, with an impact on quality of care. However, the nursing management, knowing its working group, was able to use strategic means, such as round tables and training sessions, focusing on the staff’s needs, aiming at making the situation experienced in this period less dense.

The nurse manager’s leadership role at that time was, in addition to clarifying doubts and uncertainties, motivating the nursing staff to use motivational tools. The nurse manager encouraged co-participation in the multidisciplinary staff, in order to acquire supplies for care, build and innovate care processes in their own and unique way. There was the involvement of feelings of individual growth and professional recognition, referring to the activities and their execution. Nursing management was able to show that each professional has an important role within the institution, through an individual’s self-realization.

## DISCUSSION

The challenges of hospital restructuring involved changes in structures and processes and health staff mobilization directly and indirectly linked to patient care with COVID-19. Four strategies have been recommended by the WHO to contain the COVID-19 pandemic: prepare and be ready; detect, prevent and treat; reduce and suppress; and innovate and improve^(11)^. Thus, the hospital reorganized care flow for patients with suspected COVID-19 infection and proposed a new internal and external flowchart for inpatients. Thus, 20 extra beds were made available for caring for critically ill patients and another 22 beds for caring for patients classified as non-critical. Similar changes were observed in China^(12)^.

In February 2020, the Chinese already knew that about 15% of patients with COVID-19 would develop severe pneumonia and that 6% would require intensive care with ventilatory support. However, only 600 beds were available in the city of Wuhan. Thus, there was a need to adapt the hospital structure, with the creation of 70 new ICU beds, and three general hospitals were quickly converted into intensive care hospitals with around 2,500 specialized beds for caring for patients with severe pneumonia due to COVID-19. These changes reflect the Chinese HCS resilience and its strategic planning to care for the most critically ill patients^(10)^. The Chinese shared, with the world, the main challenges faced in the pandemic period. The information helps other countries make decisions and contributes to HCS resilience and performance in changing structures and processes.

Care flow reorganization also contributed to reducing the hospital’s bed occupancy rate from 76% to 62%. Previous study showed the relationship between high bed occupancy rates and worse patient outcomes^(13)^. Therefore, reducing this rate is important in the current pandemic scenario, as the number of people infected with COVID-19 increases, so does the need for hospitalization and intensive care.

Regarding the challenges faced in equipment and supply acquisition, the investments made by the hospital to care for critical patients and PPE suitable for each stage of care stand out. The importance of PPE in sufficient quantity to care for patients with COVID-19 is highlighted, given that, in Italy, high rates of infection and death were recorded among health professionals due to the lack of PPE^(14)^.

The shortage of qualified personnel to care for critically ill patients and the nursing staff sizing to work in the COVID-19 unit and other sectors were other challenges to be overcome. The COVID-19 pandemic has placed an enormous burden on HCS, which will depend on a sufficient number of nurses with adequate resources to face this and future challenges arising from this crisis^(15)^. Nurses and other nursing staff members are on the front lines of fighting the pandemic and at the epicenter of the disease crisis. For these reasons, unprecedented levels of work overload are being witnessed by nursing staff as well as nurse managers and other professionals directly involved in the response to this pandemic. Nursing professionals around the world report reduced rest time and lack of support and mental healthcare, which directly reflects on professionals’ well-being on the front lines of the COVID-19 pandemic^(16)^.

Nursing workload continues to be an issue in healthcare settings, especially during the COVID-19 pandemic. Adequate staffing and resources, administrative support, and teamwork collaboration have been shown to improve patient safety^(15)^. However, the nurse will have to demonstrate a new level of leadership and be able to adapt to the constantly evolving the health center dynamics^(16)^.

Faced with the scarcity of scientific evidence to support hospital managers in decision-making in the face of the pandemic, the importance of articulating professional skills is highlighted in the assistance, technical and administrative areas, aiming at producing solutions to overcome the challenges of hospital management in the context of the COVID-19 pandemic. This challenge contributed to facing the pandemic in search of the recovery of individuals affected by the disease, in addition to minimizing the negative impacts of this scenario.

It is worth noting that, with the creation of a crisis committee among managers, the institution can develop action plans and share ideas, as well as align conduct within the institution. Managers used teamwork as a resilient action in the face of the scenario as being essential for work planning and organization. Health managers were challenged to manage institutional processes within a global crisis. Even in the face of personal fear, witnessing staff falling ill, they still needed to exercise a leadership role, trying to motivate the staff, point out ways and make each professional realize how important their role is in safe care.

Thus, it is critical to offer targeted care to nurses working on the front lines during the COVID-19 pandemic and who have a greater and more stressful workload than usual, needing institutional support, stress monitoring, meal breaks and rest to maintain their professional activity^(17,18)^.

New established workflows, in which admission and assistance processes for users suspected or diagnosed with COVID-19, as well as internal and external communication processes, risk prevention and control measures, strategic actions related to hospital logistics, supply administration and management, purchases and outsourcing, are only possible through the use of management tools, such as process mapping and the development of institutional protocols. Thus, the elaboration and implementation of these new workflows at different levels of assistance can be facilitated, contributing to the organization and optimization of activities to face the pandemic^(19)^.

In view of this, the management staff, based on literature regarding world experiences and resilient behavior, can be assertive in the actions taken to face the COVID-19 pandemic. New strategies were adopted for health staff training, staff sizing, supply acquisition for care and new internal flows.

The speed with which COVID-19 has spread worldwide has brought with it the challenges of overcoming a crisis with present and future effects. Therefore, the crisis due to the lack of health and financial staff generated by the pandemic is very worrisome, as it was already a great challenge even before the beginning of the COVID-19 pandemic. Given this, it is known that over the next five years, the global patient safety movement will have to learn from the negative and positive effects resulting from the pandemic, and it is time to build and consolidate safer healthcare systems that minimize harm to patients and healthcare professionals^(20)^.

### Study Limitations

As this is an experience report, the situations presented in this article may not reflect the reality of other services.

### Contributions to Nursing

The institution’s adoption of resilience in healthcare was a positive experience in developing the capacity to adapt and transform its structures and means of operation to provide the necessary service with safety and quality. Bearing this in mind, it is evident that health services can be quickly mobilized and transformed to meet the health need.

## CONCLUSION

In this experience report, the main challenges faced by a nurse manager facing the restructuring of a hospital service in the face of the COVID-19 pandemic were presented. The institution’s internal service flow reorganization required structural and process adaptations, including an increase in the number of ICU beds and wards. Equipment and supply acquisition for patient care was only possible due to the resilient behavior of managers involved in the process through the allocation of extraordinary institutional resources that, in addition to guaranteeing the expansion of care infrastructure and the operation of services during the period of crisis, enabled equipment supply and strategic inputs for the institution.

The adversities faced in health work during the COVID-19 pandemic made the manager nurse’s capacity for resilience, an indispensable feature for emotional health maintenance, and that of everyone involved, and for patient and healthcare professional safety.

One of the greatest challenges that the institution still faces is the front-line staff sizing, as it demands from the management staff a balance between nursing professionals’ well-being and the resources available by the institution, both financial and human. Thus, health service resilience is fundamental for hospital restructuring in the COVID-19 pandemic, however, it must consider caring for patients and healthcare professionals’ physical and mental health.

## ASSOCIATE EDITOR

Thereza Maria Magalhâes Moreira
